# Analysis of a Pharmacy Developed, Outpatient Lactation Consultant Initiated Domperidone Programme

**DOI:** 10.34763/jmotherandchild.20242801.d-23-00093

**Published:** 2024-04-19

**Authors:** Katherine Chinnery, Stephanie Wai Khuan Teoh, Tamara Lebedevs, Myra Kildunne, Nabeelah Mukadam

**Affiliations:** Pharmacy Department, King Edward Memorial Hospital, Perth, Western Australia, 6008, Australia; Breastfeeding Centre, King Edward Memorial Hospital, Perth, Western Australia, 6008, Australia

**Keywords:** Lactation, Breastmilk, Breastfeeding, Domperidone, Lactation Consultant, Pharmacy, International Board-Certified Lactation Consultants (IBCLC)

## Abstract

**Background:**

Domperidone is a commonly prescribed galactagogue used off-label for lactation insufficiency. Prescriber unfamiliarity or safety concerns can lead to therapeutic delay and potential early breastfeeding discontinuation. To facilitate access, the study site pharmacy department developed a Structured Administration and Supply Arrangement (SASA) for International Board-Certified Lactation Consultants (IBCLC) to screen and initiate domperidone using a checklist.

**Material:**

To validate a domperidone screening tool via analysis of its use and compliance, together with a staff satisfaction survey.

**Methods:**

Records were extracted from the REDCap® database for women with documented domperidone supply between 06/05/2022 and 27/01/2023 and reviewed with medical records. A staff survey was distributed assessing compliance and attitudes towards the SASA.

**Results:**

Records of supply revealed that 34% (17/50) of patients were referred to a physician, revealing a discrepancy between database documentation and checklists, as no referrals were documented. Overall staff satisfaction with the SASA was rated 4.6 out of 5. 77.7% (7/9) felt confident counselling and supplying domperidone with the SASA in place. 88.9% (8/9) felt confident using the checklist to identify the appropriateness of therapy and referral to a physician.

**Conclusions:**

The system in place allows the IBCLCs to initiate and supply domperidone in a timely manner to breastfeeding mothers with lactation insufficiency. The support tools, including domperidone SASA, REDCap® documentation database and the checklist domperidone as a Galactagogue Checklist, can be greatly appreciated by the LCs. Continued discussion with IBCLCs to refine and improve the SASA and associated education package will result in more consistent compliance.

## Introduction

Low breast milk supply is one of the most common reasons for discontinuing breastfeeding despite well-recognised benefits for both the mother and infant [1,2]. These benefits include preventing infection and necrotising enterocolitis, optimising nutrition and encouraging neurodevelopment in the infant [1,2]. Following inadequate response to first-line non-pharmacological measures, galactagogues may be trialled [3]. Domperidone has approval for use in Australia as an antiemetic and for the treatment of gastroparesis [4] and is used off-label to improve breast milk supply by increasing prolactin levels [5]. It is one of the most commonly utilised yet controversial pharmacological galactagogues due to the concerns regarding its potential for QTc interval prolongation [6,7,8,9,10].

There is low certainty evidence that the use of oral galactagogues in non-hospitalised term infants improves infant weight and milk volume, and this uncertainty of evidence affects both willingness to prescribe and public safety concerns [6,11,12,13]. Whilst there is little to no consensus on the exact regimen, most countries recommend a dose of 10–20 mg three times daily as per the EMPOWER trial for 14 days or longer [2,5, 10,11, 14,15,16,17,18,19]. Domperidone for lactation stimulation is well tolerated and has few contraindications [2,5,17,18,20]. In a 2016 Canadian study by Smolina et al., the incidence of arrhythmia hospitalisations linked to postpartum domperidone use was 21 in 22,532 women. However, all these women had a prior history of ventricular arrhythmias [21]. This suggests a role for electrocardiograms (ECGs) and/or physical exams in screening for cardiac abnormalities when commencing domperidone therapy but is not currently standard practice [10,11].

There are few studies that assess domperidone prescribing, access and stewardship. Haase et al. developed a clinical protocol to assist practitioners in determining the appropriateness of domperidone therapy for low breast milk supply [22]. This protocol included a checklist to determine precautions for commencing treatment and assess the need for referral to a senior physician. The Adelaide Mums and Babies Clinic uses a similar domperidone checklist [23]. Despite the existence of such clinical checklists, their use in facilitating and documenting patient screening, counselling and domperidone supply is vastly underutilised [12,24].

Mothers initially referred to LCs when experiencing low milk supply may be recommended domperidone. The mother must then seek a prescription from their general practitioner (GP) or specialist, who may be reluctant to prescribe domperidone due to unfamiliarity with the indication or the patient themselves [11]. This delay in accessing treatment and information from medical practitioners may discourage mothers and increase the likelihood of discontinuing breastfeeding altogether, [9] a consequence that could be avoided if appropriately trained LCs were involved in the domperidone supply. Currently, there are no studies or programmes reviewing domperidone supply for lactation stimulation by health professionals who do not normally have prescribing authority (such as LCs).

In the study site, as stated in the hospital clinical guideline,[3] the most important factors to increase milk production are the frequency of breastfeeding or milking and proper techniques, such as ensuring correct infant positioning and attachment, effective milk removal, unrestricted breastfeeding, avoiding long periods between feeds, offering both breasts and using gentle breast compression to encourage let down [3]. Domperidone may be considered following appropriate assessment and deemed an inadequate response to first-line non-pharmacological management according to the hospital guidelines [3]. In 2022, a SASA based on the Haase et al. protocol was implemented in the study hospital [22]. A SASA is a written direction that authorises a health practitioner to administer or supply medicine to any patient meeting the specified circumstances [25]. A SASA was put in place to allow clinical midwives certified as International Board-Certified Lactation Consultants (IBCLCs) at the Breastfeeding Centre within the study hospital to initiate and supply domperidone for patients deemed inadequate response from the first-line non-pharmacological management according to the hospital clinical guidelines [3]. The implementation of the SASA involved the preparation and endorsement of: (1) a hospital domperidone SASA with relevant guidelines such as dose, instruction, patient leaflet and supply quantity; (2) domperidone as a Galactagogue Checklist to assist in the assessment of the suitability of domperidone for the patient and to identify potential pre-existing medical conditions which require referral to the doctor; (3) a REDCap® database to document the supply of domperidone is documented. The SASA in place enables the IBCLCs to initiate and supply domperidone in a timely manner to breastfeeding mothers with lactation insufficiency.

## Aim

This activity aimed to assess IBCLCs’ compliance with the domperidone SASA and guidelines, validate the appropriate use of the domperidone as a Galactogogue Checklist, assess the frequency and indication of referral to a medical practitioner and assess the satisfaction of IBCLCs with the domperidone SASA.

## Material and Methods

### Setting

The 300-bed study hospital is the only tertiary obstetrics, gynaecological, and neonatal hospital in Western Australia (WA). Approximately 6000 births take place in the hospital annually, and it is the only referral centre for complex, high-acuity pregnancies in the state. The hospital has a 92-bed neonatal intensive care unit (NICU) designed to care for premature and unwell infants. The Breastfeeding Centre of WA (BFC), which is located at the study hospital, provides a state-wide helpline for public and health professionals, in-person appointments, telehealth and online antenatal and postnatal interactive classes with an IBCLC on weekdays. Inpatients with complex breastfeeding issues or mastitis readmissions are also reviewed on the wards prior to discharge.

### Data Documentation and Collection

**Figure 1.**
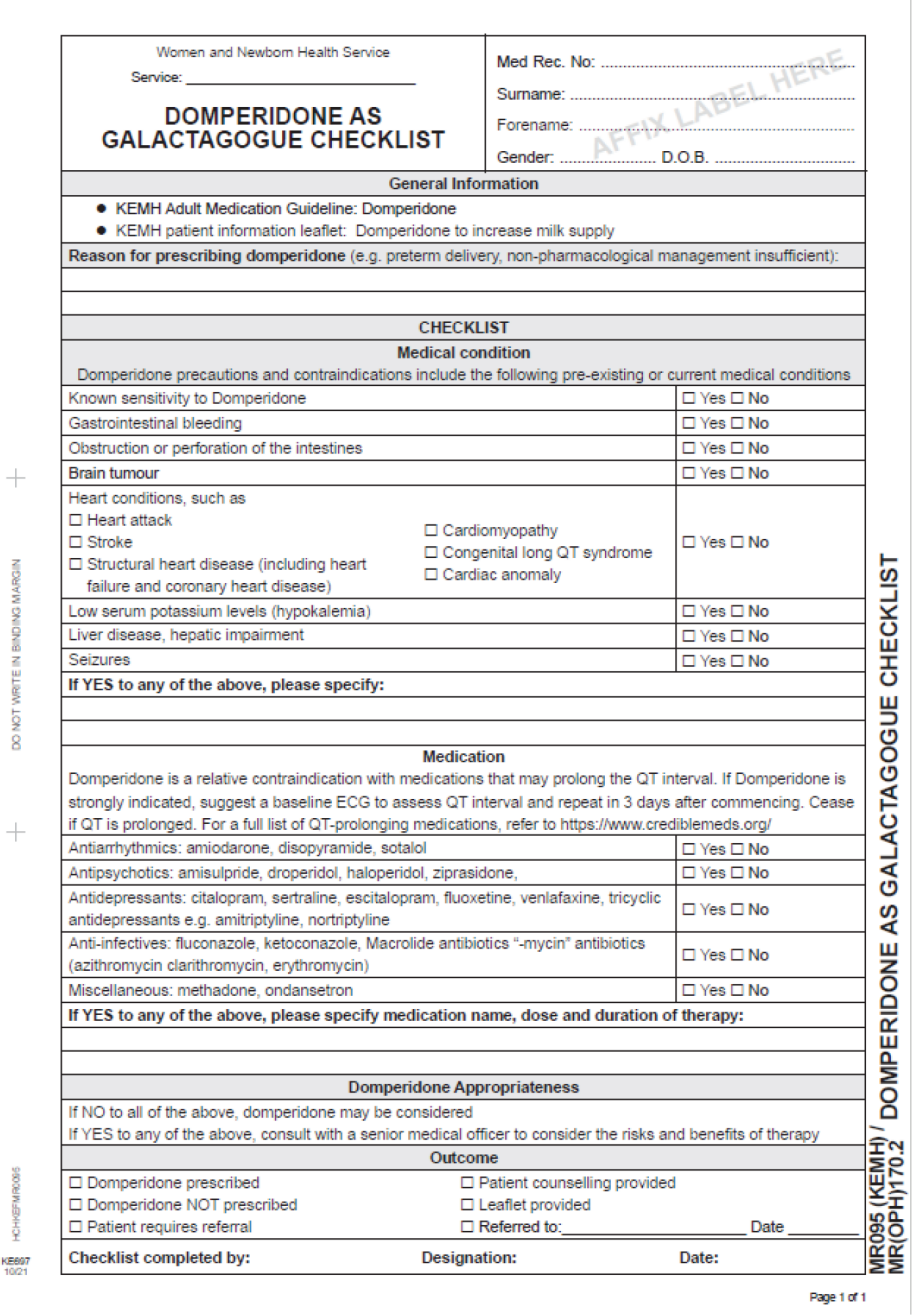
Domperidone as Galactagogue Checklist.

Upon completion of an internal training package, IBCLCs are enabled to supply 100 tablets of domperidone (Motilium®) at 10 mg three times daily (1 month supply) to eligible breastfeeding mothers with low breast milk supply following completion of a clinical checklist - domperidone as Galactagogue Checklist (Fig. 1). Referral to a medical practitioner was indicated based on patient responses to medical history as per the checklist. The supply of domperidone is documented via a REDCap® database, including information such as patient UMRN, name and date of birth, indication of screening completed, criteria for referral met and IBCLC name, employee number and site of employment. Human Research Ethics approval was gained from the Women and Newborn Health Service Quality Improvement Committee (Approval number: GEKO 49245) at the study hospital.

## Data Analysis

### Data Documented in REDCap and Checklist

Records documented on the domperidone SASA REDCap® database for the first nine months from the implementation of the initiative, from 01/05/2022 to 31/01/2023, were extracted from the database as an Excel file, deidentified and analysed. Data analysis included maternal age distribution, infant gestational age and the incidence of criteria for referral according to the SASA. Patient medical records for patients who met the inclusion criteria were reviewed to assess any documented information on the patient’s tolerance. They reported adverse effects, as well as the corresponding completed domperidone as a Galactagogue Checklist, which was also reviewed to ascertain the indication for referral.

### Staff Survey

WNHS IBCLCs were invited to participate in the satisfaction survey via Microsoft Teams, which was emailed together with the Participant Information Form. The survey was non-identifiable, with demographic data including designation and area of practice collected. Questions assessed opinions of the domperidone SASA, checklist and REDCap® database using a 5-point Likert scale (ranging from strongly disagree to strongly agree) with additional space for individualised feedback. Data was stored in a secured database folder with restricted access until the end of the study publication, and thereafter, the data was archived.

## Results

### RedCAP® Database

**Figure 2.**
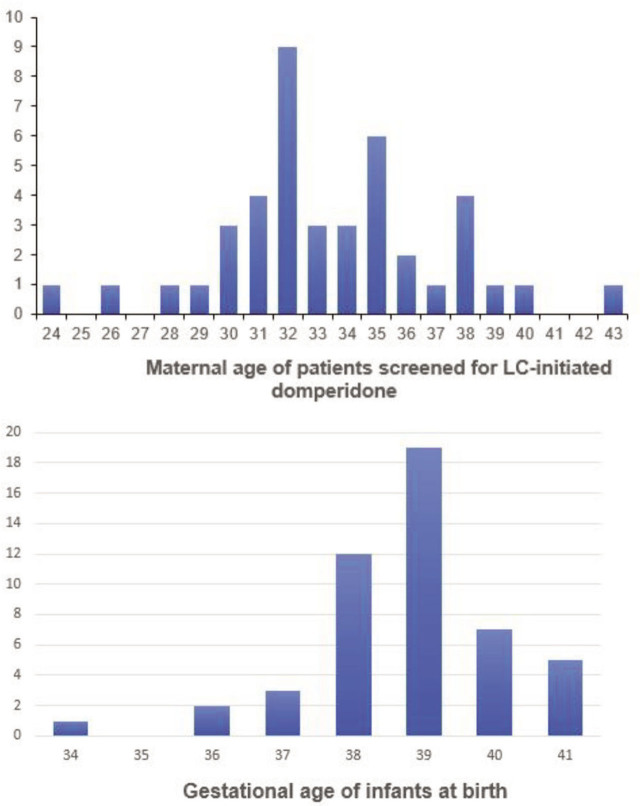
Patients screened for Lactation Consultant initiated domperidone according to maternal age and gestation age of infants at birth.

A total of fifty patients recorded via the database within the audit period, with the first entry dated 06/05/2022 and the 50th patient supplied on 27/01/2023, were analysed. This made up 2.7% (50/1835) of outpatients seen at the Breastfeeding Centre during the study period. Maternal age ranged from 24 to 43 years, with the mean age being 33.8 years (Fig. 2). All patients were recorded as having the domperidone as a Galactagogue Checklist completed with 34% (17/50) recorded as meeting the criteria for referral to a medical practitioner.

### Review of Patient Records

A total of 49 out of 50 patient medical records were reviewed, with 34 of these having completed the domperidone as a Galactagogue Checklist. No checklist revealed contraindications for receiving domperidone under the SASA, and the supply was recorded as given. Three patients had a previous history of domperidone use for lactation insufficiency. Of the records without physical checklists, only one reference to a GP referral was documented. The gestational age of infants whose mothers received domperidone ranged from 34 to 41 weeks, with the mean age being 38.7 weeks (Fig. 2). Domperidone was commenced when the infants were between 3 to 30 days old, with a median age of 11 days.

Several patient records documented additional instructions upon supply of domperidone. In one case, domperidone was supplied, but the patient was asked to delay commencing treatment until they finished a course of oral fluconazole for vaginal thrush, the duration of which was unknown. A second patient had domperidone supplied whilst taking fenugreek, a commonly used herbal galactagogue. They were encouraged to contact their GP for follow-up or if a dose increase of domperidone was required. Another patient was taking fluoxetine antenatally and ceased at 15 weeks; however, domperidone was supplied as they had no plans to restart antidepressant therapy postpartum. Finally, domperidone was supplied to one patient despite concurrent therapy with citalopram 10mg daily. An ECG was recommended for 14 days’ time with further consultation from the GP. No adverse effects to domperidone were reported. However, documentation indicated one patient did not think domperidone was helping to increase breast milk supply.

There was little to no reporting of specific follow-up instructions for domperidone therapy. Four patients had documentation of weaning (either self-initiated or practitioner-recommended), none of which had specific regimes. Three patients cancelled follow-up appointments. One patient switched to formula feeding after 2 weeks. Five patients appeared to be continuing domperidone at least one month after commencing therapy.

### Staff Survey

Nine out of fifteen WNHS IBCLCs completed the staff survey. An average rating of 4.56 out of 5 was given for overall staff satisfaction with the domperidone SASA.

**Figure 3.**
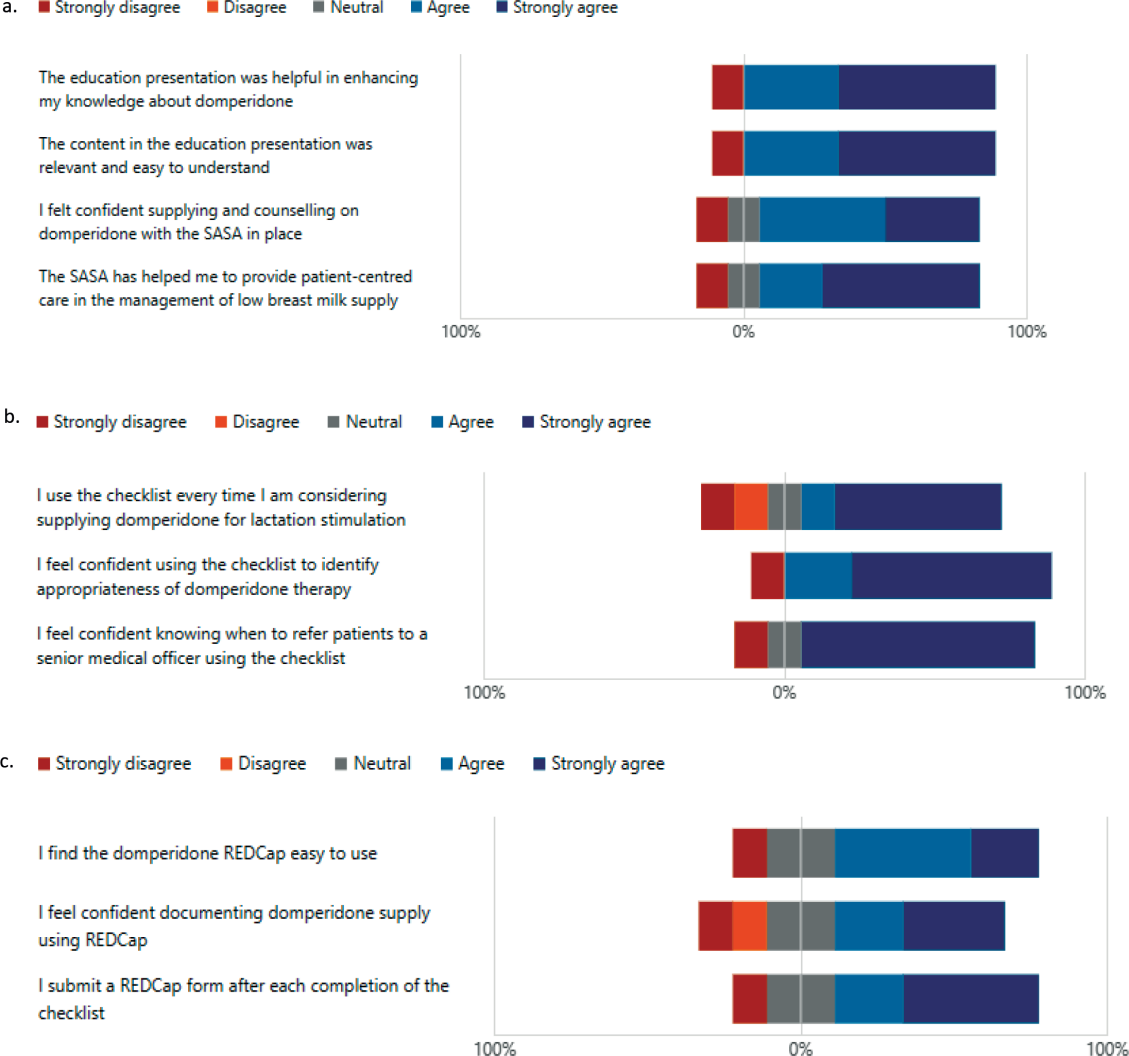
Staff agreement with statements on domperidone 3a. Staff agreement with statements on domperidone SASA 3b. Staff agreement with statements on the Domperidone as Galactagogue Checklist. 3c. Staff agreement with statements on the Domperidone SASA RedCAP.

The first set of questions concerned opinions on the SASA itself. In response to two statements regarding the effectiveness of education in preparing for domperidone supply under the SASA, eight (88.9%) agreed or strongly agreed that the presentation was helpful in enhancing knowledge and was relevant and easy to understand. One (11.1%) of the responders strongly disagreed with these statements. (Fig. 3a). One respondent also commented that “the education package was informative, explained the drug’s use, action and contraindications. Current evidence on how to titrate the dose would have been beneficial.” In response to the statement “I felt confident supplying and counselling on domperidone with the SASA in place”, seven (77.7%) agreed or strongly agreed, one (11.1%) was neutral, and one (11.1%) responder strongly disagreed (Fig. 3a).

The second set of questions concerned the use of the checklist. In response to the statement “I use the checklist every time I am considering supplying domperidone for lactation stimulation”, six (66.7%) agreed or strongly agreed, one (11.1%) was neutral, two (22.2%) disagreed or strongly disagreed (Fig. 3b). In response to the statement “I feel confident using the checklist to identify appropriateness of domperidone therapy”, eight (88.9%) agreed or strongly agreed, and one (11.1%) responder strongly disagreed. Finally, in response to the statement, “I feel confident knowing when to refer patients to a senior medical officer using the checklist”, seven (77.8%) strongly agreed, one (11.1%) was neutral, and one (11.1%) responder strongly disagreed.

The third set of questions concerned the use of a database to monitor SASA compliance. In response to the statement “I find the domperidone REDCap*®* easy to use”, two (22.2%) strongly agreed, four (44.4%) agreed, two (22.2%) were neutral and one (11.1%) of the responders strongly disagreed (Fig. 3c).

**Figure 4.**
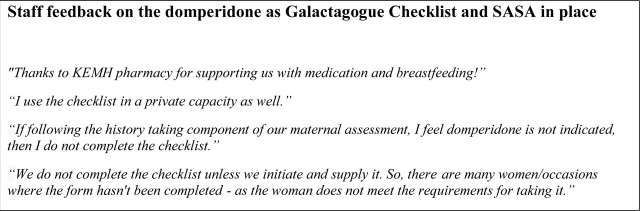
Staff feedback on the Domperidone Checklist and domperidone SASA in place.

In response to the statement “I feel confident documenting domperidone supply using REDCap*®*”, five (55.5%) strongly agreed, or agreed, two (22.2%) were neutral, one (11.1%) disagreed and one (11.1%) of responders strongly disagreed. Lastly, in response to the statement “I submit a REDCap*®* form after each completion of the checklist”, four (44.4%) strongly agreed, two (22.2%) agreed, two (22.2%) were neutral and one (11.1%) of responders strongly disagreed (Fig. 3c). At the end of the survey, a space for individualised feedback was provided for any further comments relating to the domperidone SASA (Fig. 4).

## Discussion

This is the first study and programme in Australia reviewing domperidone initiation and supply for lactation stimulation by the IBCLCs. This study evaluated and assessed compliance to and effectiveness of the SASA for IBCLC-initiated domperidone, the satisfaction of IBCLCs regarding the SASA use and the potential for IBCLC-initiated domperidone supply to be successfully established in other Australian tertiary healthcare settings.

Given the 4.56 out of 5 overall satisfaction rating and that most survey respondents strongly agreed with statements relating to the SASA structure and education, IBCLCs appeared highly satisfied with the SASA and its ability to supply domperidone directly to patients. Over half of the survey respondents strongly agreed that the domperidone as a Galactagogue Checklist aided in identifying the appropriateness of therapy and the need for referral. This suggests the checklist is sufficiently comprehensive and may be helpful in screening for the appropriateness of domperidone in other healthcare settings.

Two areas for improvement were identified regarding SASA compliance. Firstly, it was apparent that the checklist was not used with every screening of domperidone for low breast milk supply appointments, with only 55.6% of respondents answering “strongly agree” to using and completing the checklist with each domperidone screening. Multiple free-text comments indicated that IBCLCs were aware of the main contraindications for domperidone and omitted to complete the checklist if the patient did not clearly meet the criteria for supply. The checklist was, therefore, treated more as a final step prior to supplying domperidone rather than a tool to determine supply. As a result, the exact proportion of patients screened for domperidone to treat low breast milk supply is undeterminable and is likely underestimated due to non-reporting. Additionally, the frequency and most common reasons for referral (such as medical conditions and current medications) are likely not documented, as these would have been verbally discussed with the patient during the IBCLC-specific maternal assessments.

In reviewing potential reasons for lower compliance with the checklist, it was determined that the SASA does not explicitly direct that the checklist must be completed with every domperidone screening regardless of supply or non-supply. To increase the comprehensiveness of future audits, it is recommended that the SASA be updated with clearer instructions before checklist completion.

Secondly, a discrepancy between the REDCap® database reporting and physical checklists was identified. According to the database, 34% of patients were referred to GPs due to meeting the referral criteria. However, the physical checklists revealed that domperidone was supplied 100% of the time. Multiple survey comments stated that there was confusion regarding the wording of this statement on the REDCap® form, and this will be clarified. An additional education session where the updated change is publicly discussed would be helpful.

No adverse effects to domperidone or the need to cease therapy were reported. However, it was found that follow-up discussions regarding domperidone therapy were infrequently and/or vaguely documented. At least one survey respondent requested further information on how to approach weaning doses. Both IBCLCs and patients would benefit from clearly written instructions for weaning domperidone under the SASA, as seen in the Haase et al. protocol [23]. Several patient notes documented increases in milk volume exceeding 90mL, which is in line with current evidence [1, 14, 16,17,18]. However, this documentation was not uniform and outside the scope of this current audit as domperidone efficacy was not assessed.

Following this audit, the SASA and education package would be revised to include: (1) Clarification on the circumstances in which the domperidone as a Galactagogue Checklist should be completed (i.e. with every discussion of domperidone for lactation insufficiency); (2) Rewording of the statement on REDCap® from “criteria for referral” to “meets criteria for referral to senior physician” or similar to avoid staff confusion and encourage consistent reporting of referrals; (3) Standardised frameworks to document follow-up of domperidone therapy, including but not limited to weaning instructions, adverse effects and efficacy.

### Limitations

One of the study’s limitations was the non-inclusion of patient feedback in SASA implementation. Whilst several studies highlight the anxieties faced when mothers are delayed in receiving domperidone treatment for low breast milk supply, [9,11] this audit cannot conclusively state that domperidone supply under the SASA is preferred from a patient perspective.

## Conclusion

The system in place allows the IBCLCs to initiate and supply domperidone in a timely manner to breastfeeding mothers with lactation insufficiency. The support tools, including domperidone SASA, REDCap® documentation database and the checklist domperidone as a Galactagogue Checklist, can be greatly appreciated by the IBCLCs. Continued discussion with IBCLCs to refine and improve the SASA and associated education package will result in more consistent compliance. There is potential for the programme to be expanded to other sites with updates to the SASA requiring the use of the checklist with each screening and encouraging increased follow-up documentation of adverse effects, duration and weaning of domperidone therapy. Other health professionals may use this model of care to advance the scope of practice in other fields.

### Key Points

- This study describes the initiation and supply of domperidone by the International Board-Certified Lactation Consultants (IBCLCs) via a SASA.
- The system in place allows the IBCLCs to initiate and supply domperidone in a timely manner to breastfeeding mothers with lactation insufficiency.
- This study evaluates compliance with and effectiveness of the SASA in place and the satisfaction of IBCLCs regarding its use.
- Records were extracted from the database for women with documented domperidone supply and reviewed with medical records. A staff survey was distributed assessing compliance and attitudes towards the initiative.
- The study showed the initiative was greatly appreciated by the IBCLCs. The study also highlighted areas for improvement to ensure consistent compliance.
- This is the first study and programme in Australia reviewing domperidone initiation and supply for lactation stimulation by IBCLCs.
